# Supplementary material for: Cryptanalysis and improved mutual authentication key agreement protocol using pseudo-identity
Source: PLoS One. 2022 Jul 28;17(7):e0271817. doi: 10.1371/journal.pone.0271817 (PMC9333255; doi:10.1371/journal.pone.0271817)
Supplement: Attachment — Submitted filename: CRYPTANALYSIS PAPER.pdf [file pone.0271817.s001.pdf]

Manuscript Number: PONE-D-22-11432

Title: Cryptanalysis and improved mutual authentication key agreement protocol using pseudo-identity

The authors have developed a mutual key agreement scheme to be used in various applications.

They have also analysed the performance of the proposed scheme using BAN logic and AVISPA tools. The authors must address the following comments before submitting the next version of the manuscript.

The abstract must contain the name of the technical algorithm which the authors had used when comparing their work with the other two different schemes given in the abstract.

The introduction section must be strengthened. It must cover the scope of the proposed work and the objectives must be clearly defined.

Moreover, the proposed mutual key agreement scheme given in the manuscript must cover the following manuscripts. The following manuscript can be considered for analysis in the related work or for comparison in the performance analysis section. The titles of some notable referecnes are given below

- ✓ Secure multifactor authenticated key agreement scheme for industrial IoT.
- ✓ An efficient group key agreement protocol for secure P2P communication.
- ✓ An efficient anonymous authentication and key agreement scheme with privacy-preserving for smart cities.
- ✓ A provably secure dynamic ID-based authenticated key agreement framework for mobile edge computing without a trusted party.
- ✓ An unlinkable authenticated key agreement with collusion resistant for VANET's
- ✓ A PUF-based lightweight authentication and key agreement protocol for smart UAV networks.

Based on the above considerations, I would recommend MAJOR revision.
