# Supplementary material for: Cryptanalysis and improved mutual authentication key agreement protocol using pseudo-identity
Source: PLoS One. 2022 Jul 28;17(7):e0271817. doi: 10.1371/journal.pone.0271817 (PMC9333255; doi:10.1371/journal.pone.0271817)
Supplement: Attachment — Submitted filename: Response to Reviewers.doc [file pone.0271817.s002.doc]

PONE-D-22-11432

Cryptanalysis and improved mutual authentication key agreement protocol using pseudo-identity

**Dear editorial board of PLoS One**

Thank you for your effort to review my manuscript “PONE-D-22-11432: Cryptanalysis and improved mutual authentication key agreement protocol using pseudo-identity”.

I have revised the manuscript in accordance with the opinions of the editors and reviewers and am very pleased to send the revised manuscript.

Below we describe the responses to the editor and reviewers.

**Responses to the editor**

1. The manuscript has been revised to meet PLOS ONE’s style requirements as indicated by the editor.

For this, we have referred to PLOS ONE style templates at <https://journals.plos.org/plosone/s/file?id=wjVg/PLOSOne_formatting_sample_main_body.pdf> and <https://journals.plos.org/plosone/s/file?id=ba62/PLOSOne_formatting_sample_title_authors_affiliations.pdf>

2. The manuscript has been thoroughly copyedited for language usage, spelling, and grammar.

Thank you for your suggestions about AJE and Editage.

We corrected spelling and grammar errors as much as possible.

3. For this study, we did not receive any funding. So, we have included the state-“The authors received no specific funding for this work.” within our cover letter.

4. We have referred Tables 1 and 2 in the text of our revised manuscript and ensured that there was no table which we did not refer.

**Responses to the reviewer1**

1) You pointed that “The abstract must contain the name of the technical algorithm which the authors had used when comparing their work with the other two different schemes given in the abstract.” in your comments.

In our paper, we have used the experiment results of Odelu et al.’s scheme and Kilinc et al.’s scheme when comparing the computational cost of our proposed scheme with other related schemes. And we describe about this in the abstract and Section 6 of our manuscript.

2) You also pointed that “The introduction section must be strengthened.”

We added a more detailed description of the scope and purpose of the study in the introduction section. We also studied the papers you suggested and cited them and included them in the related work.

**Responses to the reviewer2**

1) You pointed that the communication was completely happened through the secure channel in user revocation phase.

If a user loses or is stolen by a malicious user, the user must revocate the already registered user at the user revocation phase.

Since the user information in the proposed protocol is stored on a smart card, we argue that the user should work on a secure channel in the revocation phase as well as in the registration phase.

2) You pointed that there was no proper explanation available about the withstanding against the forgery attack.

We considered the same type of attack as the impersonate attack and forgery attack in the previous manuscript. In this revised manuscript, we individually analysed the forgery attack resistance by distinguishing it from impersonate attack resistance. In the informal security analysis section, we analysed the resistance of the proposed scheme to forgery attacks. We describe about this in lines from 930 to 941.

3) You pointed that there was no proper justification against the man-in-the-middle attack.

We have analysed in detail the man-in-the-middle attack resistance of the proposed scheme in the revised manuscript. We focused on the fact that the man-in-the-middle attack was largely composed of the initiator impersonate attack and the responder impersonate attack, and revealed the attack resistance against it.In our protocol, the session initiator is just the user and the session responder is the server. We have analysed the resistance of our proposed scheme against the man-in-the-middle-attack in the informal security analysis section. We describe about this in lines from 904 to 918.

4) You pointed that the simulation tools were not mentioned in performance analysis.

In the previous manuscript, we used the labels we defined without numerically comparing the computational cost. In the revised manuscript, we use the experimental results of the previous researchers to calculate the computational cost more accurately, based on which we compare the computational cost with the previous schemes. In our paper, we have used the experiment results of Odelu et al.’s scheme, Kilinc et al.’s scheme and Jangirala et al’s scheme to compare the computational cost of our proposed scheme with other related schemes. We describe about it in lines from 945 to 967.

Additionally, we also compare the communication overhead with previous works and describe it in line from 968 to 980.

We hope that the revised manuscript meets the requirements of the editors and reviewers.

Thank you and best regards.

from Kyongsok Pak
